# Healthcare providers’ perspectives on family presence during resuscitation in the emergency departments of the Kingdom of Bahrain

**DOI:** 10.1186/s12873-020-00365-4

**Published:** 2020-08-31

**Authors:** Feras H. Abuzeyad, Ahmed Elhobi, Wael Kamkoum, Luma Bashmi, Ghada Al-Qasim, Leena Alqasem, Naser Mohamed Ali Mansoor, Stephanie Hsu, Priya Das

**Affiliations:** 1 Department of Emergency Medicine, King Hamad University Hospital, Building 2345, Road 2835, Block 228, P. O. Box 24343, Busaiteen, Kingdom of Bahrain; 2 Scientific Research & Development, King Hamad University Hospital, Building 2345, Road 2835, Block 228, P. O. Box 24343, Busaiteen, Kingdom of Bahrain; 3 Emergency Medicine Department–Royal Medical Services, Bahrain Defence Force, Riffa, Kingdom of Bahrain; 4 National Health Regulatory Authority, Sanabis, Kingdom of Bahrain; 5 Emergency Medicine Department, Salmanyia Medical Complex, P.O. Box 12, Manama, Kingdom of Bahrain

**Keywords:** Resuscitation, Health policy, Emergency medicine, Family presence, FPDR, Middle East, Bahrain

## Abstract

**Background:**

Worldwide, policies exist on family presence during resuscitation (FPDR), however, this is still lacking in the Gulf Corporation Countries (GCC) in general and in the Kingdom of Bahrain in particular. The aim of this study is to assess the perspectives of healthcare providers (HP) on FPDR among those working in the emergency departments (EDs) in the Kingdom.

**Methods:**

A self-administered anonymous electronic survey was collected from 146 HPs (emergency physicians and nurses) working in the three major EDs in the Kingdom of Bahrain. Besides demographic data, 18 items measuring HPs’ perceptions of FPDR were generated using the 5-point Likert scale.

**Results:**

Surveys (*n* = 146) from physicians and nurses were analysed (45.9% vs. 54.1%, respectively). There were significant differences between physicians and nurses in terms of personal beliefs, FPDR enhancing professional satisfaction and behaviour, and the importance of a support person and saying goodbye (*p* < 0.001). However, general responses demonstrated that the majority of HPs encouraged and supported FPDR, but with greater support from physicians than nurses.

**Conclusion:**

The study reflects that many HPs in EDs participated in and are familiar with FPDR, with the majority of ED physicians supporting it. Further studies should investigate the reasons for the lack of support from nurses. Results may contribute to the development of hospital ED policies that allow FPDR in the region.

## Background

According to the National Registry of Cardiopulmonary Resuscitation, about 9–11% of in-hospital cardiopulmonary resuscitations (CPR) occur in the emergency department (ED) [1]. This can be a traumatizing process for family members [2]. Family presence during resuscitation (FPDR) is a controversial circumstance that is gaining acceptance [3]. The term FPDR was first described in the early 1980s [2, 4–7], and has since been established in North America and the United Kingdom. The concept emerged later in other parts of the world in 2009 [4]. FPDR remains highly debated in clinical practice. In Europe, about 52% of countries do not practice FPDR, and on a worldwide scale, 69% do not support FPDR [2].

Although no universal definition has been agreed upon, FPDR has been defined as “the attendance of one or more family members or significant others in a location that affords visual or physical contact with the patient during invasive procedures or CPR” [5], and it is considered an essential part of patient and family-centered care [8].

FPDR has been shown to have a significant impact on the psychological experience of family members. More specifically, being present during resuscitation reduces anxiety and increases levels of satisfaction in relation to the delivered care. In addition, FPDR has not been demonstrated to contribute to resuscitation interruption [4]. Systematic reviews and meta-analysis studies highlight that FPDR does not negatively impact resuscitation outcomes and in fact improves the psychological status, satisfaction, and coping mechanism of the attending family members [2, 9].

The American Heart Association and other international organizations support FPDR [2, 5, 6, 8, 10]. In addition, many hospitals have developed written policies on FPDR [4, 6, 11, 12] and assessed its implementation. Following such policies, there were no negative effects on resuscitation management or resuscitation outcomes [12].

Current literature reveals that HPs are split between being in favour and being against FPDR, with particular differences cited between nurses and physicians. From a HP’s perspective, physicians are more resistant to the concept of FPDR compared to nurses, with main concerns regarding resuscitation quality, the potential for added conflict during resuscitation, litigations, and burdening psychological stress on family members [2, 5, 8]. Some physicians are opposed to it because of the absence of dedicated space and personnel during the resuscitation process [2]. In a study conducted in Saudi Arabia, results revealed that acute care nurses had a positive attitude about family presence. Major concerns involved patient safety, performance anxiety, and emotional impact on families [10]. Studies conducted in ED settings from Iran, Singapore, and Turkey conversely showed that most physicians and nurses are not in favour of FPDR [6, 7, 10, 13].

The presence of previous experiences with FPDR, physician seniority, specific policies supporting FPDR, and a dedicated resuscitation member influences the favorability of FPDR among HPs [2].

In order for FPDR to be considered safe and accepted, this needs specific policies, educated and trained resuscitation members, the selection of a suitable family member during resuscitation, and consideration of the social and religious aspects of the existing culture [2].

Based on the fact that the three EDs in the Kingdom of Bahrain lack policies for or against FPDR, the current study aims to investigate HP perspectives on FPDR through an electronic survey. In addition, the workforce profile of nurses and physicians working in the three EDs will help to determine if there are any demographic correlations with perceptions of FPDR. The outcomes of this study may be utilized to develop or initiate directives towards improvement in the resuscitation process.

### Ethical considerations

Ethical considerations were made according to the seven elements stated in the National Statement for Ethical Conduct of Research. The study’s scope, aims, themes, questions and methods were reviewed and provided ethical approval by the hospital’s Institutional Review Board as defined by Good Clinical Practice, compliance with National Health Regulatory Authority regulations, the country’s laws, and hospital policies governing the use of human subjects in research. The process of recruitment adhered to the ethical principles of justice and respect, as described in the study’s methods section. Consent was inclusive of all required information relevant to the proposed research in language that used appropriate vocabulary, was respectful, and relevant to the research study. Participants were given an estimated time needed to complete the survey but there was no time limit for completion. In addition, participants were notified of the choice to withdraw from the study at any point without any risks, that no personal identifiers would be collected, responses would remain anonymous, and kept with the investigators for research purposes only, and that there would be no financial involvement in the study.

Data collection, use, and management of data and information in this study were in adherence to the values of respect for human beings, research merit, integrity, justice, and beneficence. Communication of research findings to participants was not performed as the survey was anonymous. Dissemination of research outputs and outcomes would be achieved only through publication in a peer-reviewed journal in order to bring to clinicians the importance of FPDR in the management of ED patients and to encourage the development of a hospital FPDR policy. After the research project, data and information are processed according to the hospital’s Research Department guidelines where they are retained for five years to allow for potential follow up of the study population. Following the five-year period, data and information from the study will then be destroyed. At no point were researchers involved in the care of patients or responsible for family decisions in their research role.

## Methods

### Study design and population

A cross-sectional study using a self-administered randomized electronic survey was emailed to all physicians and nurses working in the EDs of the three major governmental hospitals in the Kingdom of Bahrain: Salmaniya Medical Complex, Royal Medical Services, and King Hamad University Hospital. All three hospitals are university-affiliated institutions and ethical approvals were obtained from each institution to run the study.

### Survey content and administration

The questionnaire was developed based on a survey from two studies assessing attitudes of healthcare providers on FPDR in a hospital setting [5, 14]. There were a total of 22 questions within the FPDR survey (please see Additional file 1): questions were derived from Tomlinson et al.’s study (2010) and Kianmehr et al.’s study (2010) as their questionnaires had high reliability and validity scores. A Cronbach’s α reliability coefficient was calculated on SPSS based on 18 standardized items in the questionnaire. The coefficient value was 0.789, which is an acceptable reliablity range. The questions were written and distributed in English only.

The anonymous electronic survey included three sections: 1- The first page included a digitally written participant informed consent outlining procedures, risks and benefits, and the principal investigators’ contact information at the start of the survey. At the bottom of the first page (the informed consent), participants were presented with the option to click on “I agree to the study Terms & Conditions” where they will be taken to the actual survey or “I DO NOT agree to the study Terms & Conditions” where the survey would be automatically terminated. 2- The second page covered a list of 7 demographic characteristics: age in years, gender (Male/Female), nationality (Bahraini or non-Bahraini), Profession (Physician or Nurse), Physician Level (Consultant, Senior-Registrar/Chief Resident, Senior House Officer/Resident), Level of Experience in years, and Participation in CPR (number of times per month), and 3- The third page included 22 items to identify HP perceptions of FPDR. Items 1 and 2, which were related to knowledge and experience of FPDR, had binary responses (Yes/No). Items 3 and 4 were removed after the data collection phase and prior to commencing the data analysis phase, making the total number of items measuring HP perceptions of FPDR to 20. As it was determined that the purpose of the study was to establish a policy, questions relating to the existence of a written policy allowing or prohibiting FPDR were not applicable. Items 5 to 22 were based on the 5-point Likert scale to measure the participant’s agreement or disagreement with each statement [15]. Items 9, 11, 12, 15, 17, 18, 19, 20 and 22 were reverse-coded (see Table 2).

The contact email lists of all currently working physicians and nurses in the EDs were obtained, and the Confidence Interval was set at 95% based on a population of 297. The survey and informed consent were generated on Survey Monkey. Online surveys were selected due to their low cost, anonymity and confidentiality for participants, improved efficiency and accuracy in distributing the survey and collecting data, and the option of contacting the researcher online for any questions [16]. The anonymity of participants is a priority to prevent response bias within their affiliated hospitals. Confidentiality of the collected data was secured through the use of password-protected files.

The online survey was made available for over a period of 36 days from 8th October to 12th November 2019. Reminder emails were sent throughout the data collection period to increase response rates. All answers were confidential and there was no possibility for responders to leave their names or identifiers.

### Data analysis

The data was analyzed using the SPSS statistical software version 25 to compute demographic frequencies and descriptive statistics. A confirmatory factor analysis was conducted on participant responses to a series of statements (items 5 to 22), developing codes for interpretation. Participants were asked to rate 18 statements on FPDR using a five-point Likert scale. The 18 items were subjected to principal components analysis (PCA), which included testing the data for suitability for factor analysis. A *p*-value of less than 0.05 was considered significant.

### Sample size and response rates

A priori power analysis revealed that on the basis of a medium effect size (Cohen’s *d* = 0.5) observed in past research examining the FPDR perception of healthcare providers, with a Confidence Interval (CI) of 95% and a population size of 297 ED physicians and nurses, a sample size of 168 would be needed to obtain statistical power at the recommended level [17]. The calculation was made as follows: Sample for finite population (297) with probability (*p* = 0.5) at 95% CI and 5% margin of error: n = (297 * Z2 * 0.5 * (1–0.5)/ (0.05)2)/ (297–1 + [Z2 * 0.5 * (1–0.5)/(0.05)2)] = 168.

A total of 297 surveys were administered to the ED physicians and nurses from which: 146 responded and 12 e-mails were invalid, leading to a response rate of 49% (*n* = 146), which was close to the required sample size based on p = 0.5 and CI of 95%. The sample size was considered sufficient to provide the recommended medium effect size.

## Results

### Demographic data

The demographic characteristics of the participants are shown in Table 1. Among the sample of ED HPs, 54.1% were nurses and 45.9% were physicians, and approximately 44% of the sample were Bahraini. The mean age (SD) for the participants was 37.1 years old (±9.1) with an almost equal distribution of males and females (52.1% males and 47.9% females). In terms of ED physician’s level, about 42% were senior physicians (Consultants, Senior-Registrar, or Chief Resident). Greater than 77% had 6 years of experience or more (73.1% physician and 81% nurses) and 89.8% participated in 1 or more CPR sessions per month (92.5% physicians and 87.3% nurses).

**Table 1:** Demographic data of participating healthcare providers from three National EDs in the Kingdom of Bahrain (*n* = 146)

Demographic Item	M (SD)	Range	Frequency	n
**Age (years)**				
20–25	37.1	20–55+	11 (7.5%)	146
26–35	(9.1)		53 (36.3%)	
36–45			57 (39.0%)	
46–55			19 (13.0%)	
> 55			6 (4.1%)	
**Gender**	–	–		
Male			76 (52.1%)	146
Female			70 (47.9%)	
**Nationality**	–	–		146
Bahraini			65 (44.5%)	
Non-Bahraini			81 (55.4%)	
**Profession**	–	–		
Nurse (N)			79 (54.1%)	146
Physician (P)			67 (45.9%)	
**Physician Level**	–	–		67
Consultant			10 (6.8%)	
Senior-Registrar/Chief Resident Registrar/Senior Resident			18 (12.3%) 26 (17.8%)	
Senior House Officer/Resident			13 (8.9%)	
**Level of experience (years)**				
1 to 5	> 15	1–15+	33 (22.6%) (15 N, 18 P)	146
6 to 10			37 (25.3%) (19 N, 18 P)	
11 to 15			32 (21.9%) (18 N, 14 P)	
> 15			44 (30.1%) (27 N, 17 P)	
**Participation in CPR (per month)**				
0	> 5	0–5+	15 (10.2%) (10 N, 5 P)	146
1 to 3			47 (32.1%) (17 N, 30 P)	
3 to 5			23 (15.7%) (12 N, 11 P)	
> 5			61 (41.8%) (40 N, 21 P)	

#### Knowledge and participation in FPDR

The concept of FPDR was known by 76% of the sample (82.1% physicians and 70.9% nurses) and about 64% (73.1% physicians and 55.7% nurses) had participated in CPR in the past in which a family member was present (see Table 2).

**Table 2:** Knowledge and Participation in FPDR among healthcare providers in three National EDs in the Kingdom of Bahrain (*n* = 146)

Statements	Yes		No		Chi-square
	Physician	Nurse	Physician	Nurse	
Q1. Do you know the concept of FPDR (family presence during resuscitation)?	55 (82.1%)	56 (70.9%)	12 (17.9%)	23 (29.1%)	0.08
Q2. Have you participated in CPR in which a family member was present?	49 (73.1%)	44 (55.7%)	18 (26.9%)	35 (44.3%)	0.02

In general, items [5–22] receiving a higher score on the Likert scale represented a higher agreement to FPDR by the study participants (“Strongly Agree” = 5, “Agree” = 4, “Neutral” = 3, “Disagree” = 2, Strongly Disagree = 1). Items 9, 11, 12, 15, 17, 18, 19, 20 and 22 were reverse-coded, where selecting “Strongly Agree” represented a more negative perception of FPDR and as such, the typical Likert score of 5 was recoded to be a score of 1 (see Table 3).

**Table 3:** Likert scores (in brackets) assigned to each response for individual questions

	Strongly agree	Agree	Neutral	Disagree	Strongly Disagree
Q5. Do you support implementing/producing policy allowing FPDR in your institution?	(5)	(4)	(3)	(2)	(1)
Q6. FPDR is a patient/family right	(5)	(4)	(3)	(2)	(1)
Q7. Family members should have the option to attend the CPR for adult patients	(5)	(4)	(3)	(2)	(1)
Q8. Family members should have the option to attend the CPR for pediatric patients	(5)	(4)	(3)	(2)	(1)
Q9. FPDR interfere with patient CPR (family may request to continue or to terminate CPR)	(1)	(2)	(3)	(4)	(5)
Q10. FPDR decrease family anger towards members of the code team	(5)	(4)	(3)	(2)	(1)
Q11. Family members may witness error or misinterpret some actions during resuscitation	(1)	(2)	(3)	(4)	(5)
Q12. FPDR can cause psychological stress/traumatic experience for family members	(1)	(2)	(3)	(4)	(5)
Q13. FPDR can help to grieve for family members	(5)	(4)	(3)	(2)	(1)
Q14. FPDR keeps family members updated about progress of resuscitation	(5)	(4)	(3)	(2)	(1)
Q15. FPDR need adequate space in the resuscitation room	(1)	(2)	(3)	(4)	(5)
Q16. FPDR needs to be dedicated and trained personnel to accompany family members	(5)	(4)	(3)	(2)	(1)
Q17. FPDR is stressful for members of the code team	(1)	(2)	(3)	(4)	(5)
Q18. FPDR may pose a physical threat for members of the code team	(1)	(2)	(3)	(4)	(5)
Q19. FPDR increase fear of complaints/litigations against members of the code team	(1)	(2)	(3)	(4)	(5)
Q20. FPDR may breach patient confidentiality	(1)	(2)	(3)	(4)	(5)
Q21. FPDR will motivate members of the code team to manage the patient in a more humane manner	(5)	(4)	(3)	(2)	(1)
Q22. FPDR impede training of junior staff during CPR	(1)	(2)	(3)	(4)	(5)

#### Possible confounds

Zero-order correlations were calculated for gender (male/female), nationality (Bahraini/non-Bahraini), and level of experience (years), individually against scores on items 5–22 to determine if there were any possible confounds, i.e., demographics that correlated with the survey items, making them possible “third variables” in those relationships. These correlations (see Table 4) showed nationality to covary with 8 survey items [5, 8, 10, 14–16, 19, 21] and gender to covary with item 5. This illustrates that gender may impact healthcare providers’ responses to supporting implementing/producing policy allowing FPDR in their institution. It also showed that nationality may impact participant opinions on FDPR enhancing professional satisfaction and behaviour, the importance of a support person and saying goodbye, and several items in code 1 and 2 (see Table 5).

**Table 4:** Zero-Order Correlations of Gender and Nationality with FPDR Survey Items 5–22

	Q5	Q6	Q7	Q8	Q9	Q10	Q11	Q12	Q13	Q14	Q15	Q16	Q17	Q18	Q19	Q20	Q21	Q22
**Gender**	**0.00**	0.07	0.20	0.15	0.40	0.77	0.16	0.91	0.21	0.95	0.22	0.48	0.35	0.44	0.89	0.67	0.17	0.95
**Nationality**	**0.00**	0.12	0.39	**0.00**	0.47	**0.01**	0.56	0.06	0.12	**0.02**	**0.04**	**0.05**	0.12	0.06	**0.03**	0.20	**0.04**	0.45

*Significant correlations are in bold

**Table 5:** Distribution of responses of healthcare providers to each of the 17 questions

Question no.	Likert score 1 No. of response (%)	Likert score 2 No. of response (%)	Likert score 3 No. of response (%)	Likert score 4 No. of response (%)	Likert score 5 No. of response (%)	Likert score Mean ± SD	Chi-square*
**Code 1: Personal Beliefs**							
Q5.	30 (20.5%) P: 7 (10.4%) N: 23 (29.1%)	28 (19.2%) P: 5 (7.5%) N: 23 (29.1%)	35 (24.0%) P: 19 (28.4%) N: 16 (20.3%)	40 (27.4%) P: 27 (40.3%) N: 13 (16.5%)	13 (8.9%) P: 9 (13.4%) N: 4 (5.1%)	2.85 ± 1.28	**0.00**
Q6.	15 (10.3%) P: 4 (6.0%) N: 11 (13.9%)	28 (19.2%) P: 9 (13.4%) N: 19 (24.1%)	44 (30.1%) P: 21 (31.3%) N: 23 (29.1%)	49 (33.6%) P: 26 (38.8%) N: 23 (29.1%)	10 (6.8%) P: 7 (10.4%) N: 3 (3.8%)	3.07 ± 1.10	0.10
Q7.	28 (19.2%) P: 7 (10.4%) N: 21 (26.6%)	37 (25.3%) P: 12 (17.9%) N: 25 (31.6%)	26 (17.8%) P: 11 (16.4%) N: 15 (19.0%)	48 (32.9%) P: 32 (47.8%) N: 16 (20.3%)	7 (4.8%) P: 5 (7.5%) N: 2 (2.5%)	2.79 ± 1.23	**0.001**
Q8.	25 (17.1%) P: 3 (4.5%) N: 22 (27.8%)	41 (28.1%) P: 11 (16.4%) N: 30 (38.0%)	21 (14.4%) P: 11 (16.4%) N: 10 (12.7%)	42 (28.8%) P: 28 (41.8%) N: 14 (17.7%)	17 (11.6%) P: 14 (20.9%) N: 3 (3.8%)	2.90 ± 1.31	**0.00**
Q13.	11 (7.5%) P: 3 (4.5%) N: 8 (10.1%)	33 (22.6%) P: 9 (13.4%) N: 24 (30.4%)	43 (29.5%) P: 19 (28.4%) N: 24 (30.4%)	51 (34.9%) P: 32 (47.8%) N: 19 (24.1%)	8 (5.5%) P: 4 (6.0%) N: 4 (5.06%)	3.08 ± 1.05	**0.017**
**Code 2: Impact on Professional Practice and Performance**							
Q9.	30 (20.5%) P: 14 (20.9%) N: 16 (20.3%)	52 (35.6%) P: 27 (40.3%) N: 25 (31.6%)	35 (24.0%) P: 16 (23.9%) N: 19 (24.1%)	23 (15.8%) P: 9 (13.4%) N: 14 (17.7%)	6 (4.1%) P: 1 (1.5%) N: 5 (6.3%)	2.47 ± 1.11	0.516
Q11.	25 (17.1%) P: 13 (19.4%) N: 12 (15.2%)	84 (57.5%) P: 38 (56.7%) N: 46 (58.2%)	16 (11.0%) P: 7 (10.4%) N: 9 (11.4%)	13 (8.9%) P: 5 (7.5%) N: 8 (10.1%)	8 (5.5%) P: 4 (6.0%) N: 4 (5.1%)	3.00 ± 1.16	0.943
Q12.	45 (30.8%) P: 17 (25.4%) N: 28 (35.4%)	80 (54.8%) P: 39 (58.2%) N: 41 (51.9%)	11 (7.5%) P: 6 (9.0%) N: 5 (6.3%)	5 (3.4%) P: 4 (5.1%) N: 1 (1.3%)	5 (3.4%) P: 1 (1.5%) N: 4 (5.1%)	1.94 ± 0.91	0.241
Q15.	39 (26.7%) P: 23 (34.3%) N: 16 (20.3%)	82 (56.2%) P: 38 (56.7%) N: 44 (55.7%)	14 (9.6%) P: 5 (7.5%) N: 9 (11.4%)	6 (4.1%) P: 1 (1.5%) N: 5 (6.3%)	5 (4.1%) P: 0 (0.0%) N: 5 (6.3%)	2.01 ± 0.92	**0.048**
Q17.	44 (30.1%) P: 18 (26.9%) N: 26 (32.9%)	80 (54.8%) P: 39 (58.2%) N: 41 (51.9%)	14 (9.6%) P: 8 (11.9%) N: 6 (7.6%)	5 (3.4%) P: 2 (3.0%) N: 3 (3.8%)	3 (2.1%) P: 0 (0.0%) N: 3 (3.8%)	1.92 ± 0.85	0.402
Q18.	33 (22.6%) P: 16 (23.9%) N: 17 (21.5%)	80 (54.8%) P:32 (47.8%) N: 48 (60.8%)	17 (11.6%) P: 8 (11.9%) N: 9 (11.4%)	16 (11.0%) P: 11 (16.4%) N: 5 (6.3%)	0 (0)	2.11 ± 0.88	0.205
Q19.	31 (21.2%) P: 16 (23.9%) N: 15 (19.0%)	78 (53.4%) P: 30 (44.8%) N: 48 (60.8%)	21 (14.4%) P: 12 (17.9%) N: 9 (11.4%)	15 (10.3%) P: 9 (13.4%) N: 6 (7.6%)	1 (0.7%) P: 0 (0.0%) N: 1 (1.3%)	2.16 ± 0.90	0.261
Q20.	21 (14.4%) P: 10 (14.9%) N: 11 (13.9%)	72 (49.3%) P: 30 (44.8%) N: 42 (53.2%)	35 (24.0%) P: 14 (20.9%) N: 21 (26.6%)	17 (11.6%) P: 12 (17.9%) N: 5 (6.3%)	1 (0.7%) P: 1 (1.5%) N: 0 (0.0%)	2.35 ± 0.89	0.172
Q22.	22 (15.1%) P: 12 (17.9%) N: 10 (12.7%)	68 (46.6%) P: 32 (47.8%) N: 36 (45.6%)	33 (22.6%) P: 13 (19.4%) N: 20 (25.3%)	17 (11.6%) P: 10 (14.9%) N: 7 (8.9%)	6 (4.1%) P: 0 (0.0%) N: 6 (7.6%)	2.43 ± 1.01	0.112
**Code 3: Enhances Professional Satisfaction and Behavior**							
Q10.	18 (12.3%) P: 4 (6.0%) N: 14 (17.7%)	36 (24.7%) P: 15 (22.4%) N: 21 (6.6%)	28 (19.2%) P: 8 (11.9%) N: 20 (25.3%)	56 (38.4%) P: 34 (50.7%) N: 22 (7.8%)	8 (5.5%) P: 6 (9.0%) N: 2 (.5%)	3.00 ± 1.16	**0.004**
Q21.	14 (9.6%) P: 2 (3.0%) N: 12 (15.2%)	27 (18.5%) P: 8 (11.9%) N: 19 (24.1%)	32 (21.9%) P: 11 (16.4%) N: 21 (26.6%)	57 (39.0%) P: 36 (53.7%) N: 21 (26.6%)	16 (11.0%) P: 10 (14.9%) N: 6 (7.6%)	3.23 ± 1.16	**0.001**
**Code 4: Importance of a Support Person and Saying Goodbye**							
Q14.	11 (7.5%) P: 2 (3.0%) N: 9 (11.4%)	25 (17.1%) P: 6 (9.0%) N: 19 (24.1%)	24 (16.4%) P: 11 (16.4%) N: 13 (16.5%)	73 (50.0%) P: 39 (58.2%) N: 34 (43.0%)	13 (8.9%) P: 9 (13.4%) N: 4 (5.1%)	3.36 ± 1.10	**0.013**
Q16.	2 (1.4%) P: 0 (0.0%) N: 2 (2.5%)	10 (6.8%) P: 3 (4.5%) N: 7 (8.9%)	16 (11.0%) P: 4 (6.0%) N: 12 (15.2%)	83 (56.8%) P: 34 (50.7%) N: 49 (62.0%)	35 (24.0%) P: 26 (38.8%) N: 9 (11.4%)	3.95 ± 0.87	**0.001**

*Significant Chi-Square test scores are in bold. P = Physician, N = Nurse

### Factor analysis

Principal components analysis revealed the presence of four components with eighteen values exceeding 1, explaining 25.6, 17.4, 6.4 and 6.0% of the total of 55.6% variance, respectively. The scree plot revealed a clear break after the fourth component. Hence, the four components were considered for further analysis. To aid in the interpretation of these four components, Oblimin rotation was performed. The four components were divided into four key codes by the researchers according to the statement themes: code 1- personal beliefs about FPDR, code 2- impact on professional practice and performance, code 3- enhances professional satisfaction and behaviour and code 4- the importance of a support person and saying goodbye. These codes were selected from Porter et al.’s study (2015), which was chosen due to its relevant ED setting, multi-centric approach to collecting data, and a similar cluster of questions centred around the same themes [18].

#### Code 1: personal beliefs about FPDR

For code 1 (see Table 5), a significance testing for the composite index was performed using non-parametric tests. Mann Whitney tests showed that there was a significant difference in personal beliefs about FPDR between doctors and nurses (*p* < 0.001). Physicians (53.7% vs 21.6% nurses) were more likely to support implementing/producing a policy allowing FPDR in their institution, while 58.2% of nurses disagreed with this. More physicians (62.7%) supported that family members should have the option to attend CPR for paediatric patients, while the majority of nurses (65.8%) did not support this (*p* < 0.001). This significant difference also existed when healthcare providers were asked if family members should have the option to attend CPR for adult patients (55.2% physicians in support vs. 58.2% nurses against this).

#### Code 2: impact on professional practice and performance

Regarding FPDR’s impact on professional practice and performance (see Table 5), significance testing for the composite index was performed using non-parametric tests. Mann Whitney test showed that there were no significant differences in this code among doctors and nurses (*p*-values range from 0.112 to 0.943). Mean scores reflected that healthcare providers view FPDR as not having a major negative impact on professional practice and performance. The majority *disagreed* that FPDR would: cause psychological stress for family members (85.6%), interfere with patient CPR (56.1%), cause family members to witness error or misinterpret some actions during resuscitation (72.6%), or breach patient confidentiality (63.7%).

The majority also *disagreed* that FPDR would impact members of the code team negatively, e.g., causing stress (84.9%), posing physical threat (77.4%), increasing fear of complaints/litigations against members of the code team (74,6%), or impede training of junior staff during CPR (61.7%). Only 1 of the 9 items in code 2 showed a significant difference between nurses and physicians: while the majority of physicians (91%) and nurses (76%) agreed that adequate space is not needed for FPDR (item 15), there was still a significance between the two groups (*p* = 0.048).

#### Code 3: enhancing professional satisfaction and behavior

Code 3 (see Table 5), which included item 10 and 21, demonstrated a statistically significant difference between physicians and nurses (*p* < 0.01). Physicians (59.7%) were more like to agree that FPDR decreases family anger towards members of the code team, whereas nurses were more likely to be neutral (25.3%) or disagree (24.3%) (*p* = 0.004). Additionally, 68.6% of physicians agreed that FPDR will motivate members of the code team to manage the patient in a more humane manner, whereas only 34.2% of nurses agreed (*p* < 0.001).

#### Code 4: importance of a support person and saying goodbye

There was a statistically significant difference in how physicians and nurses responded to code 4 (see Table 5), which focused on the theme of “The importance of a support person and saying goodbye” (items 14 and 16). More physicians (71.6%) than nurses (48.1%) agreed that FPDR keeps family members updated about the progress of resuscitation (*p* = 0.013). However, the majority of physicians (89.5%) and nurses (73.4%) agreed that FPDR needs dedicated and trained personnel to accompany family members (*p* < 0.001).

## Discussion

This is the first study in the Kingdom of Bahrain to provide insight into HP’s attitudes towards FPDR in emergency departments. There were significant differences between physicians and nurses in terms of personal beliefs, FPDR enhancing professional satisfaction and behaviour, and the importance of a support person and saying goodbye (*p* < 0.001). General responses demonstrated that the majority of HPs encouraged and supported FPDR with greater support from physicians than nurses.

Furthermore, there were no significant differences in distribution of opinion between physicians and nurses (*p*-values range from 0.112 to 0.943) on the impact of FPDR on professional practices and performance during resuscitation. The majority of HPs *disagreed* that FPDR would significantly impact professional practice and performance. For example, the majority *disagreed* that FPDR would: cause psychological stress for family members (85.6%), interfere with patient CPR (56.1%), cause family members to witness error or misinterpret some actions during resuscitation (72.6%), or breach patient confidentiality (63.7%). The majority of HPs also *disagreed* that FPDR would impact members of the code team negatively, e.g., causing stress (84.9%), posing physical threat (77.4%), increasing fear of complaints/litigations against members of the code team (74,6%), or impede training of junior staff during CPR (61.7%).

In terms of FPDR enhancing professional satisfaction and behaviour, results demonstrated a statistically significant difference between physicians and nurses (*p* < 0.01). Physicians (59.7%) were more like to agree that FPDR decreases family anger towards members of the code team, whereas nurses were more likely to be neutral (25.3%) or disagree (24.2%) (*p* = 0.004). Additionally, 68.6% of physicians agreed that FPDR will motivate members of the code team to manage the patient in a more humane manner (avoid black humour), whereas only 34.2% of nurses agreed (*p* < 0.001).

For code 4, “The importance of a support person and saying goodbye”, more physicians (71.6%) than nurses (48.1%) agreed that FPDR keeps family members updated about the progress of resuscitation (*p* = 0.013). However, the majority of physicians (89.5%) and nurses (73.4%) agreed that FPDR needs dedicated and trained personnel to accompany family members (*p* < 0.001) [4, 19]. Finally, nationality and, to a minor extent, gender demonstrated a significant effect on healthcare providers’ opinions to several survey items measuring HP perceptions of FPDR (see Table 4).

The current study outcomes are contrary to the outcomes of Iran, Singapore, and Turkey, where the majority of ED physicians and nurses are against FPDR [6, 7, 13]. But it is important to note that the study outcomes are in support of outcomes measured in Saudi Arabia, as well as a Needs Assessment for FPDR conducted by the American Heart Association, and a systematic review of four randomized controlled trials that looked at the psychological outcomes of FPDR on family relatives [10, 20, 21].

In Iran, a descriptive study was conducted among four hospitals measuring family and nursing perceptions of FPDR. Around 57% of female family members felt that it was a right to experience FPDR as it allowed them to observe everything and worry less. Regarding nurses, 62.5% disagreed with implementation of FPDR for adult patients, citing that family members become distressed and may prolong the resuscitation effort. Only nurses with prior education of FPDR were open to implementation [6]. In Singapore, a survey was performed amongst healthcare providers in a university-affiliated hospital. Approximately 71% of healthcare providers stated that relatives should not be present during CPR due to the possible interruption of patient care, wellbeing of the relatives, limited physical space, and resources [13]. In Turkey, a literature review was performed for five articles relating to family presence during cardiopulmonary resuscitation. The study concluded that Healthcare professionals were not in support of family presence, and those who had taken part in family presence during cardiopulmonary resuscitation had negative experiences [7].

A study in Saudi Arabia looked at the attitudes of acute care nurses’ toward FPDR in the largest hospitals in the five geographic regions of Riyadh City. Nurses were specifically recruited from the ED, Intensive Care Unit, and Critical Care Units. Results indicated that nurses had a positive attitude about family presence [10]. The American Heart Association published A Needs Assessment on FPDR, which was conducted via a survey sent to members of a Cardiothoracic Intensive Care Unit code team including physicians, nurses, and allied healthcare professionals. Up to 72% endorsed utilizing a family facilitator role to support the practice of FPDR [20]. A Cochrane systematic review of four randomized control trials was conducted to investigate if FPDR decreases post-traumatic stress disorder-related symptoms including signs of depression/anxiety. Evidence indicated overall positive results on the psychological outcomes of family relatives and that it does not affect healthcare professionals, the morbidity, or mortality of the patients negatively [21]. Our findings in this study add to the growing body of literature that looks at healthcare provider attitudes regarding FPDR.

### Limitations

One of the main limitations of this study can be attributed to the study data collection measure of administering surveys, where self-reported measures are subject to response biases. Based on the disagreement between physicians and nurses towards supporting FPDR, it would have been helpful to include an open-ended question (a statement that cannot be answered with a “yes” or “no” or be scored on a Likert scale). This will provide better insight on the reasons for or against FPDR by nurses. The sample also represents HP’s perspectives from the three EDs in governmental hospitals, whereas those from the private sector were not represented. Also, there were no questions to evaluate cultural differences and religious beliefs that might play a part in forming opinions on FPDR philosophy, which affects the face and content validity (i.e., the extent to which the measure covers all aspects of the concept it is measuring) of the current survey when measuring HP perceptions of FPDR.

## Conclusion

The concept of FPDR is familiar to many HPs in the EDs of the Kingdom of Bahrain, and a significant percentage of HPs have participated in CPR in the presence of a family member, despite the lack of policies allowing or forbidding FPDR. However, this study demonstrates that the majority of ED physicians support FPDR, whereas an equal percentage of ED nurses are against it. HPs agreed that FPDR does not affect the code team in many ways, but there was a discrepancy in viewing how the family members will benefit from attending the process with higher support from physicians than nurses. HPs also highly supported the allocation of a dedicated liaison person to accompany family members during CPR, and with such results, it is encouraging that FPDR policy would be supported by staff and can be established and implemented in the ED’s. Since the decision to implement FPDR should be a team decision that is well-planned, it would be valuable to explore potential reasons documented in the literature (e.g., barriers, self-efficacy, health beliefs, and subjective norms) behind ED nurses not supporting the concept of FPDR [22].

## Supplementary information

**Additional file 1.** Healthcare Provider Perspectives on Family Presence During Resuscitation in the Emergency Departments of the Kingdom of Bahrain. FPDR Informed Consent and Full Survey used for this research study.

## Data Availability

○ The datasets used and/or analysed during the current study are available from the corresponding author on reasonable request. ○ All data generated or analysed during this study are included in this published article.

## Abbreviations

CPR: Cardiopulmonary resuscitation
ED: Emergency Department
FPDR: Family Presence during Resuscitation
GCC: Gulf Corporation Countries
HP: Healthcare Providers
PCA: Principal components analysis
SD: Standard Deviation
