# Supplementary material for: Healthcare providers’ perspectives on family presence during resuscitation in the emergency departments of the Kingdom of Bahrain
Source: BMC Emerg Med. 2020 Aug 31;20:69. doi: 10.1186/s12873-020-00365-4 (PMC7460739; doi:10.1186/s12873-020-00365-4)
Supplement: Supplementary file 1 — Additional file 1. Healthcare Provider Perspectives on Family Presence During Resuscitation in the Emergency Departments of the Kingdom of Bahrain. FPDR Informed Consent and Full Survey used for this research study. [file 12873_2020_365_MOESM1_ESM.docx]

**INFORMED CONSENT**

| **PROJECT INFORMATION** | |
| --- | --- |
| Research Project Title | **Healthcare Providers Perspectives on Family Presence During Resuscitation in the Emergency Departments of the Kingdom of Bahrain** |
| Principal Investigator | Dr. Feras H. Abuzeyad |

The purpose of this research study is to evaluate Healthcare Provider (Physicians and Nurses) perspectives on Family Presence During Resuscitation (FPDR) in the Emergency Departments of the Kingdom of Bahrain and clarify how the concept of FPDR is accepted in the healthcare field, especially in the emergency medicine community.

You will be asked to fill a questionnaire. There is no financial compensation for your participation in this research. All information will be anonymous and kept with the investigators for research purposes only.

You are free to choose whether or not to participate in this study. There will be no penalty or loss of benefits to which you are otherwise entitled if you choose not to participate. You will be provided with any significant new findings developed during the course of this study that may relate to or influence your willingness to continue participation.

In the event you decide to discontinue your participation in the study: Please notify (Dr. Feras Abuzeyad, feras.abuzeyad@khuh.org.bh) of your decision so that your participation can be orderly terminated.

In addition, your participation in the study may be terminated by the investigator without your consent if disorderly conduct is observed or if intentionally false information is provided in order to alter the results of the study.

For the purpose of this questionnaire, the following definition will be used:

**Family Presence During Resuscitation (FPDR) – The presence of family in the patient care area, in a location that affords visual or physical contact with the patient during resuscitation events in patient triaged with level 1 emergency**

**N.B. Level-1 Emergencies include but are not limited to: Airway Compromise, Inadequate Breathing, Profound Shock, Unresponsiveness and Cardio-Pulmonary Arrest**

1. **SIGNATURE OF PARTICIPANT**

I understand the procedures and conditions of my participation described above. My questions have been answered to my satisfaction, and I agree to participate in this study.

**I agree to the study Terms & Conditions**

**I DO NOT agree to the study Terms & Conditions**

1. **Age** (years):  20-25  26-35  36-45  46-55  >55
2. **Gender**:  Male Female
3. **Nationality:**  Bahraini  Non Bahraini

If Non Bahraini, please specify _________________________

1. **Profession**: Physician Nurse

1. **Physician Position:** Consultant Senior-Registrar/Chief Resident Registrar/Senior Resident Senior House Officer/Resident
2. **Level of Experience** (years): 1 to 5  6 to 10  11 to 15  >15
3. **Participation in CPR** (per month)**:** 0 1 to 3  3 to 5  >5

|  | | | | Yes | No |
| --- | --- | --- | --- | --- | --- |
| Q1. Do you know the concept of FPDR (family presence during resuscitation)? | | | |  |  |
| Q2. Have you participated in CPR in which a family member was present? | | | |  |  |
| Q3. Do you have written policy in your department/hospital allowing FPDR? | | | |  |  |
| Q4. Do you have written policy in your department/hospital prohibiting FPDR? | | | |  |  |
|  | Strongly agree | Agree | Neutral | Disagree | Strongly disagree |
| Q5. Do you support implementing/producing policy allowing FPDR in your institution |  |  |  |  |  |
| Q6. FPDR is a patient/family right |  |  |  |  |  |
| Q7. Family members should have the option to attend the CPR for adult patients |  |  |  |  |  |
| Q8. Family members should have the option to attend the CPR for pediatric patients |  |  |  |  |  |
| Q9. FPDR interfere with patient CPR (family may request to continue or to terminate CPR) |  |  |  |  |  |
| Q10. FPDR decrease family anger towards members of the code team |  |  |  |  |  |
| Q11. Family members may witness error or misinterpret some actions during resuscitation |  |  |  |  |  |
| Q12. FPDR can cause psychological stress/traumatic experience for family members |  |  |  |  |  |
| Q13. FPDR can help grieving for family members |  |  |  |  |  |
| Q14. FPDR keeps family members updated about progress of resuscitation |  |  |  |  |  |
| Q15. FPDR need adequate space in the resuscitation room |  |  |  |  |  |
| Q16. FPDR need dedicated and trained personnel to accompany family members |  |  |  |  |  |
| Q17. FPDR is stressful for members of the code team |  |  |  |  |  |
| Q18. FPDR may pose physical threat for members of the code team |  |  |  |  |  |
| Q19. FPDR increase fear of complaints/litigations against members of the code team |  |  |  |  |  |
| Q20. FPDR may breach patient confidentiality |  |  |  |  |  |
| Q21. FPDR will motivate members of the code team to manage the patient in a more humane manner (avoid black humor) |  |  |  |  |  |
| Q22. FPDR impedes training of junior staff during CPR |  |  |  |  |  |
